# Non-invasive quantification of age-related changes in the vertebral endplate in rats using in vivo DCE-MRI

**DOI:** 10.1186/s13018-017-0669-x

**Published:** 2017-11-09

**Authors:** Hui Li, Jia-zhi Yan, Yong-jie Chen, Wei-bo Kang, Jia-xi Huang

**Affiliations:** The Department of Orthopedics, Beijing Tiantan Hospital, Capital Medical University, Dongcheng District, Beijing, 100050 China

**Keywords:** Dynamic contrast-enhanced magnetic resonance imaging, Intervertebral disc endplate, Degenerative disc disease, Lumbar spine

## Abstract

**Background:**

Small animal models that can mimic degenerative disc disease (DDD) are commonly used to examine DDD progression. However, assessments such as histological studies and macroscopic measurements do not allow for longitudinal studies because they can only be completed after the animal is sacrificed. Dynamic contrast-enhanced MRI (DCE-MRI) may provide a reliable, non-invasive in vivo method for detecting the progression.

**Methods:**

The present study investigated the progression of changes in lumbar discs and the effect of endplate conditions on diffusion into the lumbar discs of aging sand rats after intravenous administration of gadolinium-containing contrast medium through the tail vein. Contrast enhancement was measured in the lumbar intervertebral discs on each image. The results were compared with those from conventional histological characterizations.

**Results:**

T2-weighted images revealed that with aging, the shape of L3–L4, L4–L5, L5–L6, and L6–S1 nucleus pulposus (NP) became irregular, while the mean areas, signal intensities, and T2 values of the NP were significantly decreased. Each of the observed disc changes demonstrated a progressive increase in phase during 2-min scout scans. Post-contrast MRI showed impaired endplate nutritional diffusion to the disc with aging, enhancement was significantly greater in young animals than in old animals. Endplate calcification or sclerosis was histologically confirmed; histologic score was correlated with the age. We found the histological score of the endplate negatively corresponded to the DCE-MRI results.

**Conclusions:**

DCE-MRI studies offer a non-invasive in vivo method for investigating the progress of diffusion into the discs and the functional conditions of the endplate. We conclude that quantitative DCE-MRI can identify the severity of disc degeneration and efficiently reflect the progression of vertebral endplate changes in the aging sand rat lumbar spine via the NP contrast enhancement patterns.

## Background

Intervertebral disc degeneration (IDD) is one of the most common causes of low back pain, and the vertebral endplate plays an important role in disc degeneration. Recent research results have shown that nutrient diffusion through the vertebral endplate into the disc depends on fluid flow. With increasing age, the blood supply to the vertebral endplate decreases, so endplate degeneration decreases nutrient diffusion from the vertebral body into discs, leading to further disc degeneration [1]. Calcification or sclerosis of the cartilage endplate, followed by its replacement by bone, can block the diffusion of nutritional material into the disc, thereby contributing to disc degeneration [2]. Thus, experimental studies in the vertebral endplate are commonly employed to study IDD [3].

Histological studies and macroscopic assessments remain the “gold standard” for evaluating IDD progression using animal experiments. Additionally, MRI can provide important information about the severity of a degenerative condition [4]. Rajasekaran et al. found a significant correlation between total endplate score and IDD, but that method is too complicated, requiring six MRIs for 12 h [5]. Improvement is necessary, as most animal experiments can only be completed after animal sacrifice. Recent advancements in DCE-MRI have made this technique feasible for a non-invasive in vivo longitudinal quantification of intervertebral disc changes in both normal and experimental animal models. DCE-MRI, which utilizes high-field magnetic systems and high-resolution magnet systems, can detect significant changes in the signal enhancement time course in the region of interest (ROI) of intervertebral discs by using the contrast agent gadopentetate dimeglumine [6, 7].

## Methods

### Animals

To investigate the age-related changes in the vertebral endplate, DCE-MRI and histology were used to study three groups of different ages of sand rats. A total of 18 sand rats, 6 per group, were used in the study: (1) young rats, 3 months of age, (2) middle-aged rats, 6 months of age, and (3) old rats, 9 months of age. For DCE-MRI, 4 lumbar intervertebral discs (IVDs) per rat at the L1–S1 level were analyzed. For histological analysis, the L5–L6 disc and endplate were collected.

### Ethical permissions

All animal experiments were approved by the Animal Ethical Committee of the Neurosurgical Institute of Beijing, The Capital Medical University (No.201601006).

### Micro-MRI procedures and evaluation

**Fig. 1:**
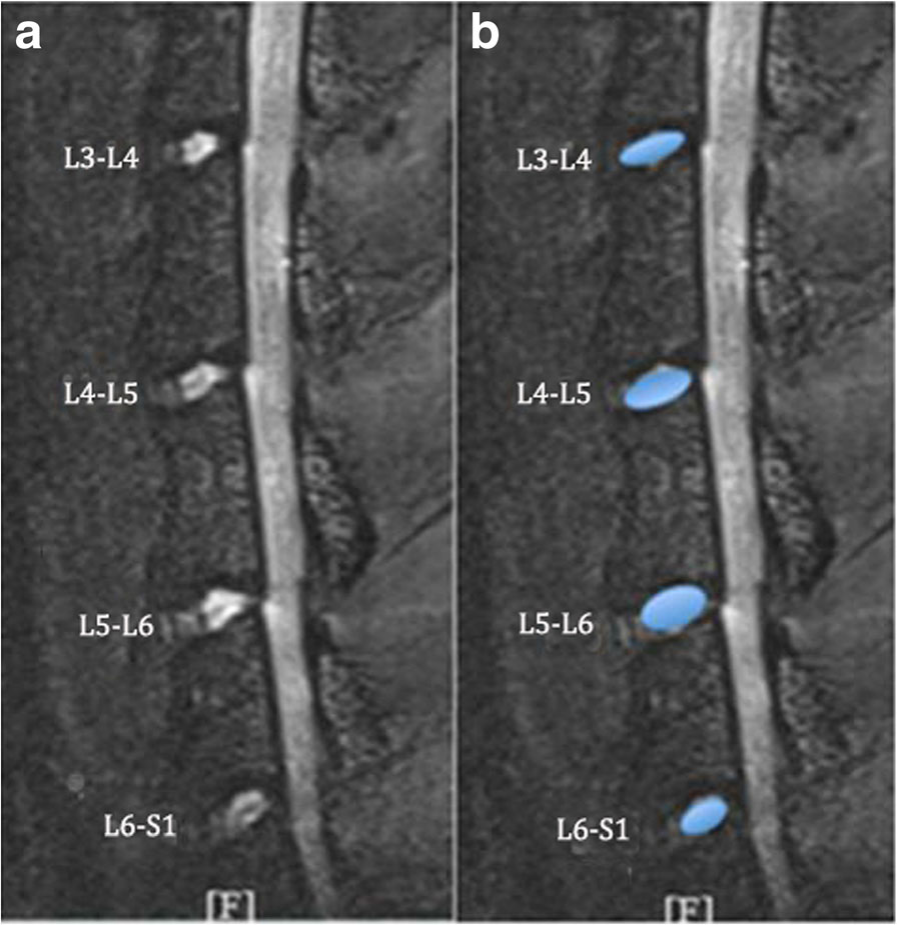
Computer screen captures showing a raw, T2-weighted magnetic resonance image of a rat lumbar spine (**a**) and the same image with NP outlined to define ROI (**b**).The measured NP area and signal intensity are shown (L3–L4 represent the intervertebral disc between the third and the fourth lumbar vertebra, and so on)

7.0-T micro-MRI was performed on a BioClinScan Animal MRI System (Siemens, Germany) with a maximum gradient strength of 290 mT/m. All rats were anesthetized using sodium pentobarbital (40 mg/kg, i.p.) before the scan, and the tail vein was cannulated with a heparinized catheter. Then, rats were placed on the examining bed in the prone position. Utilizing the iliac crest as the anatomical landmark, the region of the rat lumbar spine was covered by a surface coil that was fixed with surgical tape. First, a series of sagittal T2-weighted scans were obtained to ascertain the optimal position of the rat lumbar vertebral region for imaging, and the surface coil was repositioned if the images were not ideal. After obtaining satisfactory sagittal midsection scans, a sagittal T2-weighted scan and a sagittal T2 mapping scan were performed. The software associated with Bruker MRI scanners was used for the T2 mapping generation process. A color map was generated from the resulting T2 mapping scans of different lumbar levels. A global color scale reflecting the T2 values was applied. Regions of interest (ROIs) were placed only on the NP of the obtained sagittal midsection images. For each scan, ROIs of four consecutive intervertebral lumbar discs were selected for measurements of area and signal intensity values (Fig. 1a, b). The technique was performed by two independent blinded observers (YJC, JXH).

### DCE-MRI data processing

DCE-MRI was performed using the following parameters: TR, 2.87 ms; TE, 0.69 ms; slice thickness, 0.5 mm; imaging frequency, 300.429036; echo number, 1; and magnetic field strength, 7.05568. The contrast medium was Gd-DTPA, and a quick injection of 0.3 mmol/kg was performed manually, followed by injection of 0.5 ml of normal saline via the heparinized catheter. The time required for the scan was approximately 2 min. ROIs were placed on the NP of the sagittal midsection images. For each scan, four consecutive intervertebral lumbar discs were chosen for DCE-MRI measurements. DCE-MRI data from each disc were analyzed using the custom programmed software associated with the radiological workstation, and contrast enhancement was calculated to generate a dynamic MRI enhancement curve. And according to the previous reference, the contrast enhancement of different curves were calculated [8]. The technique and the calculation were performed by two independent blinded observers. The investigator who analyzed the enhancement data was blinded to the animal status.

### Histological analysis

The lumbar spines were removed from the rats after DCE-MRI, and the L5–L6 discs together with intact adjacent vertebral body bone were fixed in 10% neutral buffered formalin for 1 week, decalcified with EDTA and embedded in paraffin. Serial 5-μm-thick histologic slices were stained with hematoxylin and eosin (HE) and Safranin O/Fast Green, followed by qualitative analysis under a light microscope. Then, each sample was assessed and scored by two experienced pathologists independently who were blinded to the group assignments. The morphology and cellularity of the endplate disruption was analyzed using the histological grading score proposed by Boos et al. [9]. An increase in the score (ranging from 0 to 18) reflected a more severe endplate degeneration.

### Statistical analysis

The data are expressed as the mean ± standard deviation. Standard analysis of variance (ANOVA), followed by post hoc test, and analysis of covariance procedures were used to evaluate significant differences between groups (the area and signal intensity values of the NP, the T2 values of the NP). Pearson correlation coefficients were calculated to determine the correlation between changes in enhancement percentage and histologic scores in the same and different ages. Statistical significance was defined as *P* ≤ 0.05. All analyses were performed with SPSS 19.0 (SPSS Inc., Chicago, IL).

## Results

### Area and signal intensity values of NP T2-weighted images

**Fig. 2:**
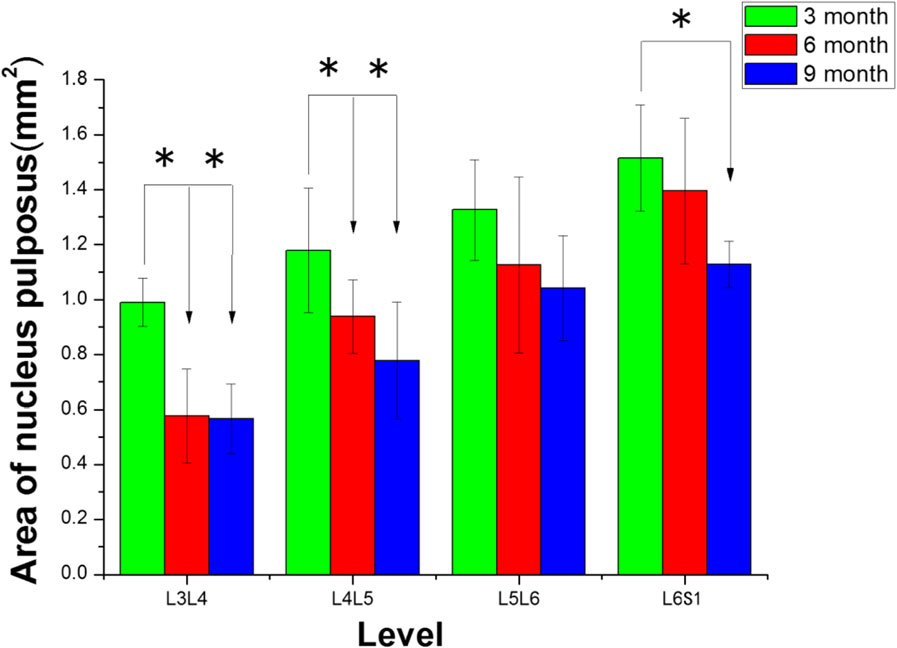
The nucleus pulposus area in different levels of the T2-weighted magnetic resonance images. *Indicates significant differences between different months, *P* < 0.05. No significant differences were observed in the intervertebral discs of different levels between 6- and 9-month-old groups (L3–L4 represent the intervertebral disc between the third and the fourth lumbar vertebra, and so on)

**Fig. 3:**
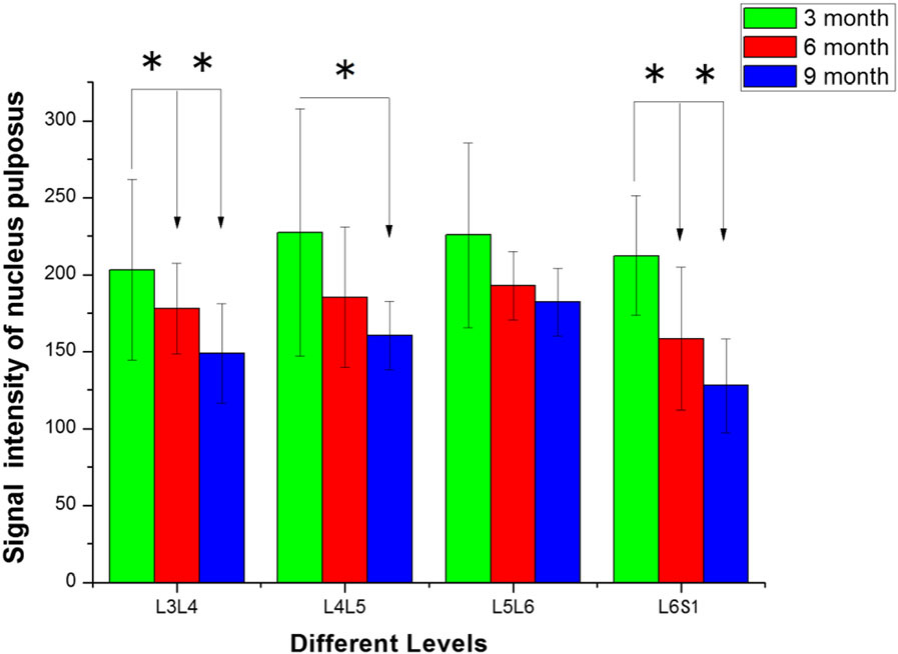
The signal intensity of the nucleus pulposus in different levels of the T2-weighted magnetic resonance images. *Indicates significant differences between different months, *P* < 0.05. No significant differences were observed in the intervertebral discs of different levels between 6- and 9-month-old groups (L3–L4 represent the intervertebral disc between the third and the fourth lumbar vertebra, and so on)

Representative serial MRI scans of the lumbar spine of one sand rat are shown in Fig. 1, consisting of T2-weighted, sagittal plane images of the L3–L4, L4–L5, L5–L6, and L6–S1 discs (Fig. 1a, b). Progressive decreases in NP area and signal intensity are apparent at 3, 6, and 9 months for each of the four discs of the different lumbar levels (L3–L4, L4–L5, L5–L6, and L6–S1). Further qualitative MRI observations are provided in Figs. 2 and 3 **.** The value for the L3–L4 NP area in 3-month-old rats was significantly greater than those in 6- and 9-month-old rats (*P* < 0.05, for both, Fig. 2); the value for the L4–L5 NP area in 3-month-old rats was significantly higher than those in 6- and 9-month-old rats (*P* = 0.049 and *P* = 0.003, respectively, Fig. 2); the value for the L6–S1 NP area in 3-month-old rats was significantly higher than that in 9-month-old rats (*P* = 0.005, Fig. 2). The values for the L3–L4 and L4–L5 NP signal intensity in 3-month-old rats were significantly higher than that in 9-month-old rats (*P* = 0.041, *P* = 0.05, respectively, Fig. 3); the value for the L6–S1 NP signal intensity in 3-month-old rats was significantly higher than those in 6- and 9-month-old rats (*P* = 0.031, *P* < 0.05, respectively, Fig. 3). There was no statistically significant difference in mean NP area and NP signal intensity in the intervertebral discs of different levels between 6- and 9-month-old rats.

**Fig. 4:**
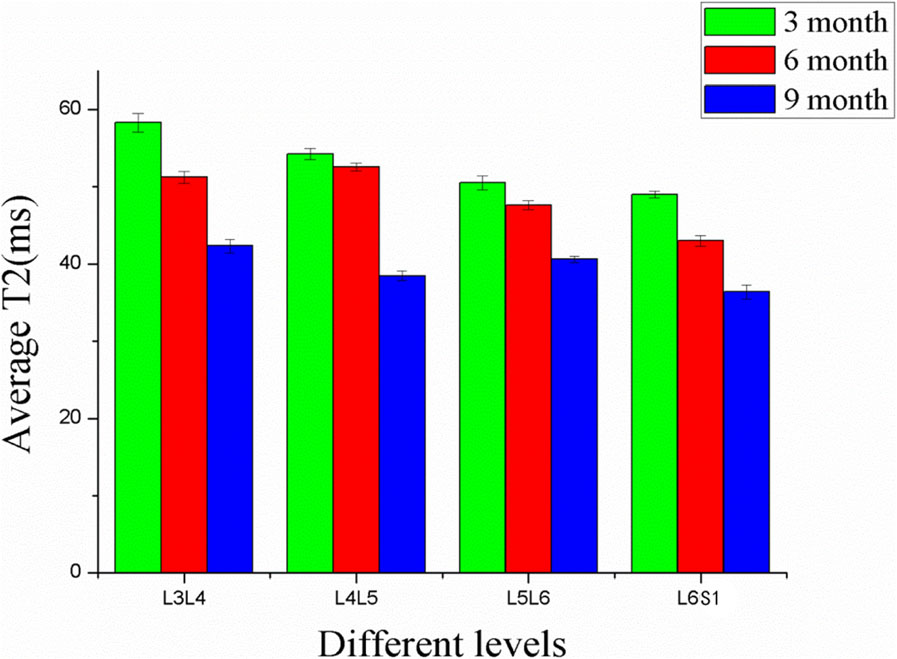
Measured T2 values from regions of interest (ROIs) in the nucleus pulposus plotted over the L3–L4 to L6–S1 disc levels from sagittal mid-axial images of rats of different ages. Comparison of T2 values between the different lumbar levels revealed differences statistically with age (*P* < 0.05, respectively) (L3–L4 represent the intervertebral disc between the third and the fourth lumbar vertebra, and so on)

**Fig. 5:**
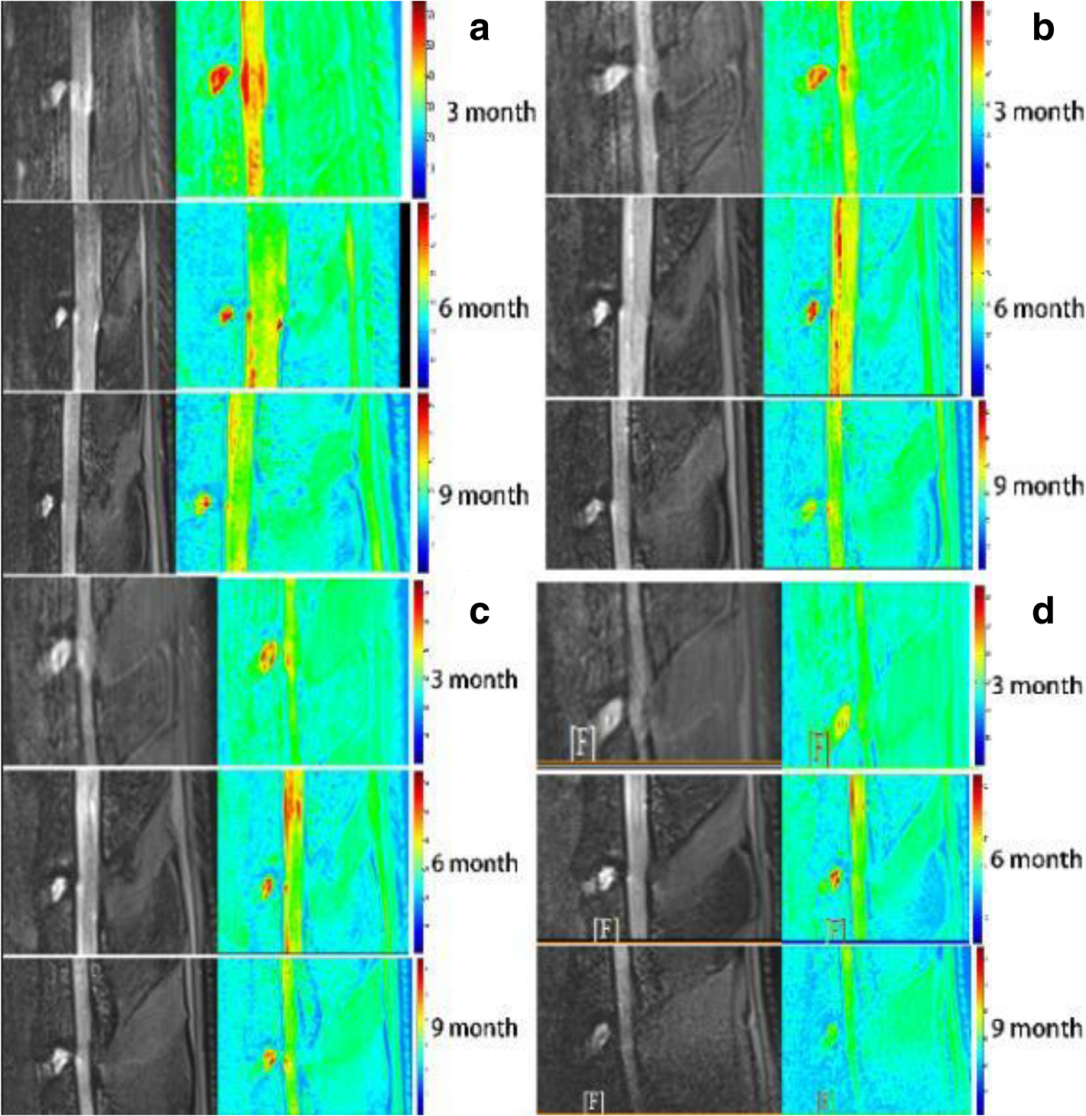
Sagittal T2-weighted images (gray images) and corresponding T2 maps (colored images) of discs with different levels. Relaxation times (ms) are displayed on the right of each T2 map image. **a** Intervertebral disc in L3–L4. **b** Intervertebral disc in L4–L5. **c** Intervertebral disc in L5–L6. **d** Intervertebral disc in L6–S1

In the 3-month-old rats, peak T2 values were concentrated in the NP, as shown by the red color. With increasing age, the NP was dominated by lower values, represented by the green and yellow colors on the T2 mapping. With increasing age, the color-coded T2 mapping showed lower NP T2 values, and the NP T2 values in the same lumbar levels were significantly lower (Figs. 4 and 5). There was statistically significant difference in mean NP T2 values in different lumbar discs at either time point (*P* < 0.05).

### DCE-MRI analysis

**Fig. 6:**
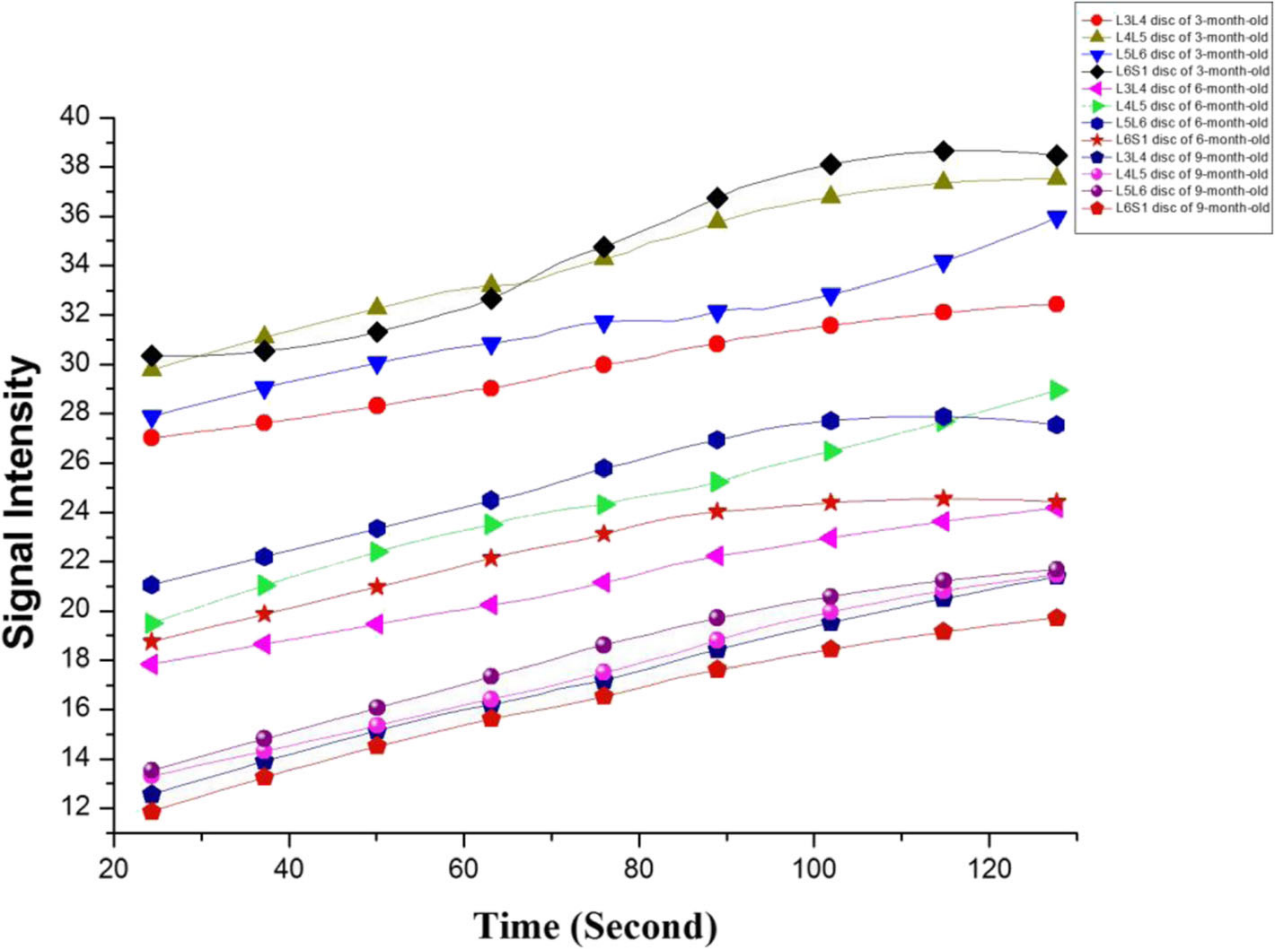
Dynamic MRI enhancement curve of different lumbar levels in different ages. Disc enhancement patterns show progressive increase within 2 min. Similar enhancement curve is seen in lumbar levels with different ages (L3–L4 represent the intervertebral disc between the third and the fourth lumbar vertebra, and so on)

The post-contrast MRI images clearly depicted the L3–S1 lumbar discs, allowing the ROIs to be placed over the four intervertebral discs, and the progress of contrast enhancement in the discs was visible in the images obtained after the intravenous contrast agent was administered. The contrast enhancement slowly diffused into the NP over time. Because the signal of the ROIs was homogeneous, consistent measurements could be obtained from the four discs. The contrast enhancement patterns differed among the lumbar levels in animals of the same age, and with aging, the NP signal intensity of the same lumbar levels was clearly diminished, with the corresponding time-intensity curve showing an obvious decrease in diffusion into the disc due to the degeneration of the endplate with aging (Fig. 6). Enhancement percentage of the NP was shown in Table 1. Enhancement percentage of the L3–L4 disc was higher in 3-month-old groups than 6-month-old groups (54.54 ± 5.81% vs 44.14 ± 8.35%, *P* < 0.05); enhancement percentage of the L6–S1 disc were higher in 3-month-old groups than 6-month-old groups (43.56 ± 12.95% vs 29.26 ± 5.04%, *P* < 0.05) and 9-month-old groups (43.56 ± 12.95% vs 25.43 ± 6.85%, *P* < 0.05).

**Table 1:** Changes in Enhancement Percentage at Center of Disc and histologic scores of endplate in different age groups in rats

Age group	Discs in L3–L4	Discs in L4–L5	Discs in L5–L6	Discs in L6–S1	Histologic score (0–18)
3-month-old	54.54 ± 5.81	47.69 ± 13.36	48.79 ± 11.45	43.56 ± 12.95	2.67 ± 0.52
6-month-old	44.14 ± 8.35*	44.84 ± 6.21	43.65 ± 9.92	29.26 ± 5.04*	9.50 ± 1.05*,**
9-month-old	40.96 ± 4.57	36.45 ± 6.12	34.29 ± 7.13	25.43 ± 6.85*	14.17 ± 1.72*

Values are expressed as the mean ± SD. Enhancement Percentage = (the maximum signal intensity postenhancement (SImax) − the signal intensity from baseline (SIbase))/SIbase × 100 (L3–L4 represent the intervertebral disc between the third and the fourth lumbar vertebra, and so on)

*P < 0.05 compared with 3-month-old groups

**P < 0.05 the histologic changes for the endplate of 6-month-old compared with 9-month-old group

### Histological studies

**Fig. 7:**
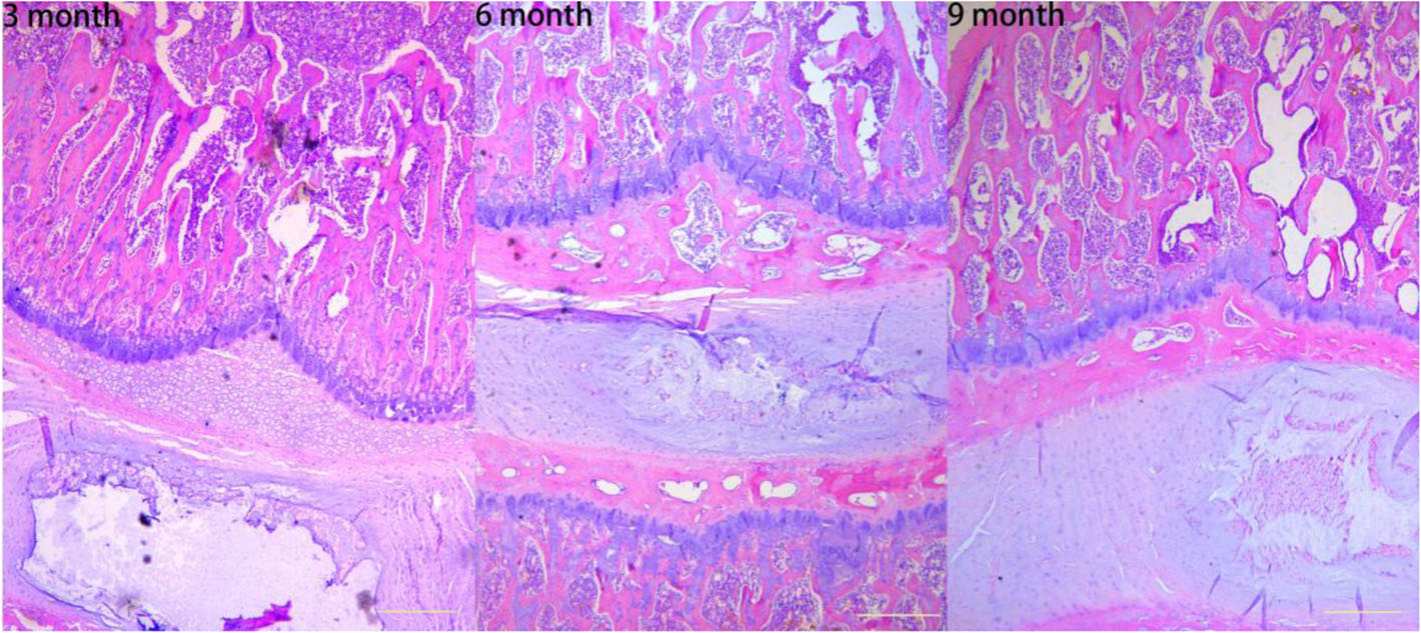
Representative histologic sections (H&E staining) of the disc and endplate (original magnification, ×10). **a** Young group. **b** Middle-aged group. **c** Old group. With aging, rats show loss of cellularity in the endplate, and the articular cartilage undergoes endochondral ossification and is replaced by bone

**Fig. 8:**
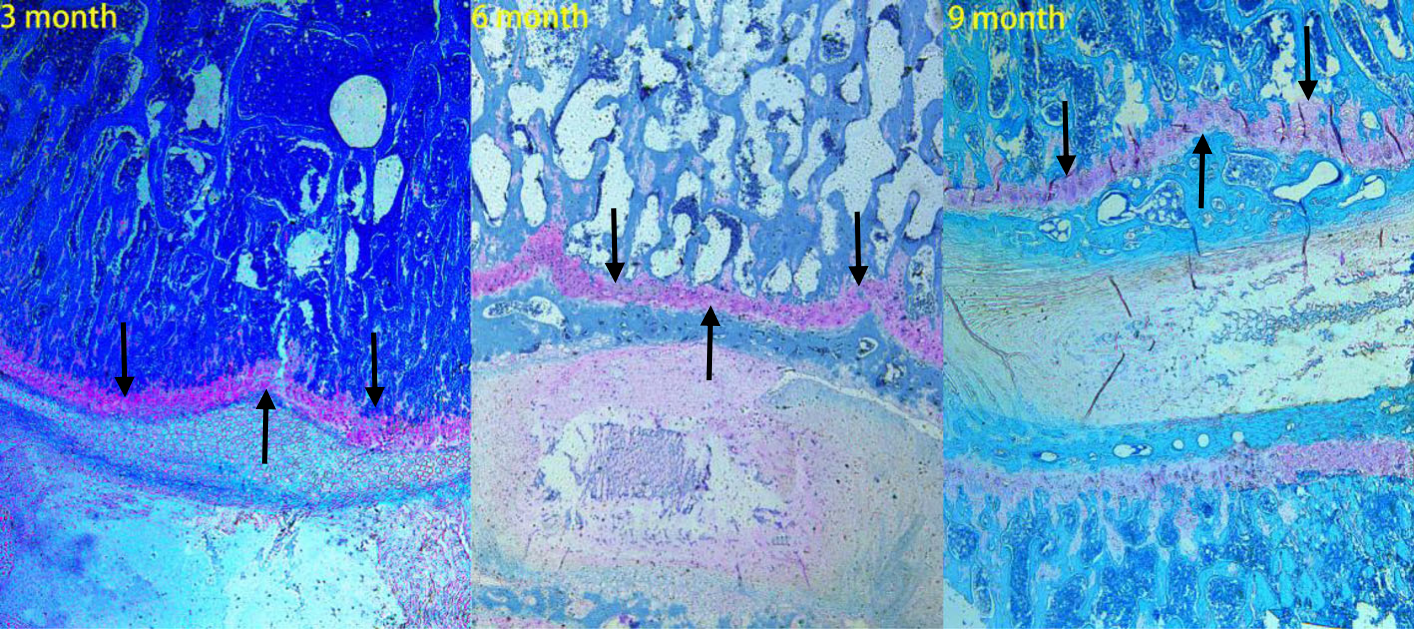
Representative histologic sections (Safranin O/Fast Green stain) of the disc and endplate (original magnification, ×10). **a** Young group. **b** Middle-aged group. **c** Old group. With aging, the concentration of sulfated glycosaminoglycans (sGAG, arrows) was decreased and resorption of the articular cartilage was replaced by bone in the endplate

Figure 7 shows representative sagittal histologic sections (HE staining) and Fig. 8 (Safranin O/Fast Green staining) of the disc and endplate of different ages (a: young group; b: middle-aged group; c: old group). We found that with aging, rats show loss of cellularity in the endplate and increases in fibroblasts, and the cartilage undergoes endochondral ossification and is gradually resorbed and replaced by bone, producing the increased endplate bone mass present in the older groups. According to the histological grading scale, the degeneration endplate scores in 3-month-old groups differed significantly from those in 6-month-old groups (2.67 ± 0.52 vs 9.50 ± 1.05, *P* < 0.05) and 9-month-old groups (2.67 ± 0.52 vs 14.17 ± 1.72, *P* < 0.05). The degeneration endplate scores in 9-month-old groups differed significantly from that in 6-month-old groups (14.17 ± 1.72 vs 9.50 ± 1.05, *P* < 0.05) (Table 1).

We found the histological score of the endplate negatively corresponded to the DCE-MRI results. There was a significant correlation between the changes in Enhancement Percentage at Center of Disc and histologic scores of endplate in 3-, 6-, and 9-month-old groups (*r* = − 0.929; *r* = − 0.942; *r* = − 0.882, respectively). Also, the correlation was significant between 3- and 9-month-old groups (*r* = − 0.815), 3- and 6-month-old groups (*r* = − 0.926), and 6- and 9-month-old groups (*r* = − 0.980) (Table. 2).

**Table 2:** Pearson’s correlation between the changes in Enhancement Percentage at Center of Disc and histologic scores of endplate in same and different age groups in rats

Age group	Correlation (*r*)	*P* value
3-month-old	− 0.929	0.007
6-month-old	− 0.942	0.005
9-month-old	− 0.882	0.020

## Discussion

The vertebral endplate is an important part of the intervertebral disc. Aging of the IDD is characterized by decreased vascularity and water content of the nucleus, changes in the collagen content, and structural changes of the cartilage endplate [10]. MRI has become the gold standard for assessment of the intervertebral disc. Modic et al. have described three types of endplate changes [11, 12]. However, it is quite challenging to produce images of cartilaginous endplate because it requires ultrahigh resolution due to its thinness, and it is therefore difficult to show the correlation between disc degeneration and endplate permeability [13].

In the present study, a grading system on the basis of serial DCE-MRI was used to investigate the changes of the vertebral endplate function. The aims were thus to detect the changes in the signal enhancement time course in the region of interest (ROI) of intervertebral discs and to analyze the correlation between the vertebral endplate function and disc degeneration. Rajasekaran et al. previously noted that a pattern with decreased diffusion indicates a decreased nutritional supply to the disc [14], which indicates that the endplate nutritional pathway is gradually affected and declines with age, leading to degenerative disc changes. Our results indicate that the age-related endplate degeneration can also be reflected by DCE-MRI.

Although histological assessments remain the gold standard for the evaluation of degenerative disc disease (DDD) progression in animal studies, they require consecutive and numerous sections to obtain typical information about the disc and endplate, and histological assessments can be completed only after the animal is sacrificed. Our results show that DCE-MRI studies offer a non-invasive in vivo method for investigating the progression of diffusion into the discs and the functional conditions of the endplate. The degenerative endplate changes can be reflected in vivo by evaluating the contrast enhancement patterns of the disc in the aging sand rat model, and the DCE-MRI results correspond to the severity of the endplate degeneration. Compared to conventional MRI, DCE-MRI provides pronounced dynamic contrast enhancement of the disc, with the following advantages: (1) it is a noninvasive method; (2) under general anesthesia, the animals can be subjected to in vivo examinations; (3) operating this system is simple, with a short learning curve; and (4) DCE-MRI can provide direct information regarding dynamic images of the physiological status of the ROIs.

Our histological analysis showed obvious degenerative endplate changes, including calcification or sclerosis of the endplate, as well as the loss of sGAG (sulfated glycosaminoglycans) and these results were consistent with the micro-MRI findings. These histological results confirm that the endplate degenerated with age, since changes in the structure of the endplate and changes in the endplate matrix components due to calcification or sclerosis can reduce blood flow and the amount of solute. For example, the contrast agent Gd-DTPA diffuses into the disc, eventually leading to a decrease in enhancement and diffusion, which can be confirmed by the DCE-MRI. And according to the histological measurements, we believe that DCE-MRI can provide a reliable, non-invasive, in vivo method for detecting endplate degeneration with age in animal models.

### Limitations

There were some limitations of this study. Firstly, the conclusions derived from the relatively small numbers of rats may require careful consideration of the assumptions. Secondly, although we chose animals of the same age and size for each of the age groups, the accuracy and reliability of data could be further increased by using larger animals or human participants. Similarly, we evaluated the age-related changes in the vertebral endplate using three groups of animals at different ages, without continuous observation of rats of the same age and size.

## Conclusions

Our study demonstrated the dynamic function activity change of the vertebral endplate in aging sand rats by using DCE-MRI analyses. With increasing age, the endplate gradually becomes sclerotic or ossific, and the endplate nutritional pathway is degraded, which thereby influences Gd-DTPA diffusion into the NP, eventually leading to changes in dynamic MRI enhancement patterns. We found the histological score of the endplate negatively corresponded to the DCE-MRI results. DCE-MRI is a reliable and non-invasive method for investigating the degeneration of discs and the functional condition of the endplate.

## Abbreviations

DCE-MRI: Dynamic contrast-enhanced MRI
DDD: Degenerative disc disease
HE: Hematoxylin and eosin
IDD: Intervertebral disc degeneration
NP: Nucleus pulposus
ROI: Region of interest
sGAG: Sulfated glycosaminoglycans
